# Does Vaccination Increase the Risk of Autism Spectrum Disorder?

**DOI:** 10.7759/cureus.27921

**Published:** 2022-08-12

**Authors:** Shaza A Mohammed, Shriya Rajashekar, Suganya Giri Ravindran, Meghana Kakarla, Musa Ausaja Gambo, Mustafa Yousri Salama, Nathalie Haidar Ismail, Pardis Tavalla, Pulkita Uppal, Pousette Hamid

**Affiliations:** 1 Internal Medicine, California Institute of Behavioral Neurosciences & Psychology, Fairfield, USA; 2 Pediatrics and Child Health, California Institute of Behavioral Neurosciences & Psychology, Fairfield, USA; 3 Internal Medicine/Family Medicine, California Institute of Behavioral Neurosciences & Psychology, Fairfield, USA; 4 Research, California Institute of Behavioral Neurosciences & Psychology, Fairfield, USA; 5 Medicine, California Institute of Behavioral Neurosciences & Psychology, Fairfield, USA; 6 Neurology, California Institute of Behavioral Neurosciences & Psychology, Fairfield, USA

**Keywords:** measles mumps rubella (mmr), immunization, vaccination, autism spectrum disorder, autism

## Abstract

Autism spectrum disorder (ASD) is a developmental disorder that can cause significant social, communicative, and behavioral difficulties. With autism rates rising dramatically in recent years, researchers and concerned parents have theorized the causes of autism, and the subject has received much attention. Is the high rate of autism now due to increased diagnosis and reporting, changing autism definitions, or a rise in the number of people with ASD? People started to blame vaccines as a cause of the increased number of people with ASD. Vaccines and their connection to autism have been the subject of continuous debate. Some parents are concerned that vaccines, particularly the measles-mumps-rubella (MMR) vaccine and preservatives used in other childhood vaccines, may play a role in developing autism in their children. This systemic review explores the link between vaccination and autism in children. We conducted a literature search using PubMed and Google Scholar. We included papers written in the English language from 1998 to 2022, conducting human research that examines the relationship between vaccination and the development of autism using appropriate quality assessment tools. Two reviewers independently reviewed the content of the included studies. In total, 21 studies were deemed eligible.

## Introduction and background

The incidence of autism spectrum disorder (ASD) has risen substantially. This rise has sparked widespread public concern regarding the causes and prevention of the condition. The prevalence of ASD among children aged six to 11 years was 3 per 10,000 in 1991-1992 which increased to 52 per 10,000 in 2001-2002 [1].

Understandably, parents of children with the condition are often angry, feeling guilt, searching for causes, and asking themselves, “Why has this happened?” Many parents blamed themselves, claiming that the problem may be due to dangerous behavior during pregnancy, advanced age at conception, or a genetic element. A narrative that blames an external aspect, on the contrary, appears to be more comfortable; vaccines were the ideal target for their rage and frustration [2].

Wakefield et al. [3] published a report in 1998 describing 12 cases of widespread developmental slowdown linked to gastrointestinal (GI) system symptoms and developmental delay, a fair amount of which occurred shortly after the administration of measles, mumps, and rubella (MMR) vaccine. The theory presented in this case series was that a new variety of ASD was developing and linked to the MMR vaccine; this raised concerns among parents regarding the MMR vaccine’s safety and vaccination in general [4,5]. Moreover, a few studies have correlated the number of vaccines added to the children’s immunization schedule with the prevalence of ASD diagnosis. Mercury toxicity and modification in immune system function have been the subject of numerous investigations [6]. Following the article by Wakefield et al. on the MMR vaccination-autism link, there has been an upsurge in the antivaccine attitude and vaccine hesitancy in the United States. A lack of readiness to embrace immunization, as shown with pertussis immunization in many countries in the 1970s and 1980s and MMR immunization in the United Kingdom and the United States, resulted in the re-emergence of vaccine-preventable diseases. In the aftermath, acceptability improved [5]. The ideas in the report continue to raise anxiety and challenge vaccine acceptance among parents [6,7].

This systemic review aims to determine any relationship between vaccination and ASD development. We will review multiple articles on vaccination/MMR/ASD and understand their correlation.

## Review

Methodology

We conducted our systematic review utilizing the Preferred Reporting Items for Systematic Reviews and Meta-Analysis (PRISMA) guidelines [8].

Database

We started our research on November 15, 2021. We used PubMed and Google Scholar as databases for our data collection.

Search strategy

We included studies on immunization/vaccination and autism/ASD. Our search mechanism included keywords and Medical Subject Headings (MeSH). Table 1 displays the results of each search. The following keywords were used in the literature search: Autism OR autistic disorder OR echolalia OR scripting OR perseveration OR spectrum disorder OR savant OR sensory processing disorder AND Immunization OR vaccination OR MMR OR DTAP OR Varicella OR Polio OR Pcv13 AND Autism ((“Autistic Disorder/chemically induced”[Majr] OR “Autistic Disorder/etiology”[Majr] OR “Autistic Disorder/immunology”[Majr] OR “Autistic Disorder/microbiology”[Majr] OR “Autistic Disorder/statistics and numerical data”[Majr] OR “Autistic Disorder/virology”[Majr])) OR (“Autistic Disorder/chemically induced”[Mesh:NoExp] OR “Autistic Disorder/etiology”[Mesh:NoExp] OR “Autistic Disorder/immunology”[Mesh:NoExp] OR “Autistic Disorder/microbiology”[Mesh:NoExp] OR “Autistic Disorder/statistics and numerical data”[Mesh:NoExp] OR “Autistic Disorder/virology”[Mesh:NoExp])immunization (("Immunization/adverse effects”[Majr] OR “Immunization/complications”[Majr] OR “Immunization/drug effects”[Majr] OR “Immunization/injuries”[Majr])) OR (“Immunization/adverse effects”[Mesh:NoExp] OR “Immunization/complications”[Mesh:NoExp] OR “Immunization/drug effects”[Mesh:NoExp] OR “Immunization/injuries”[Mesh:NoExp]).

**Table 1:** The result of the initial search.

Keywords/MeSH keywords	Google Scholar	PubMed
Autism	1,590	9
Immunization	189,000	9
Autism OR autistic disorder OR echolalia OR scripting OR perseveration OR spectrum disorder OR savant OR sensory processing disorder AND Immunization OR vaccination	12,800	1,090

Inclusion criteria

We choose peer-reviewed papers and studies from the last five years written in the English language. We only selected systematic reviews, traditional reviews, meta-analyses, and randomized trials conducted among human subjects. All data collected were within ethical and legal standards.

Exclusion criteria

We excluded gray data and papers that focused on animals. We also excluded articles published before 1998.

Quality assessment tool

We used the following quality assessment tool to evaluate the papers utilized in this study: a Measurement Tool to Assess Systematic Reviews (AMSTAR) questionnaire for systematic reviews and meta-analysis, Cochrane risk bias assessment tools for randomized control trials, the scale for the Assessment of Narrative Review Articles (SANRA) for traditional reviews, and Newcastle-Ottawa Scale for observational studies. We discarded poor-quality studies.

Data collection

We collected the data from the selected articles (with high quality) individually.

Results

**Figure 1:**
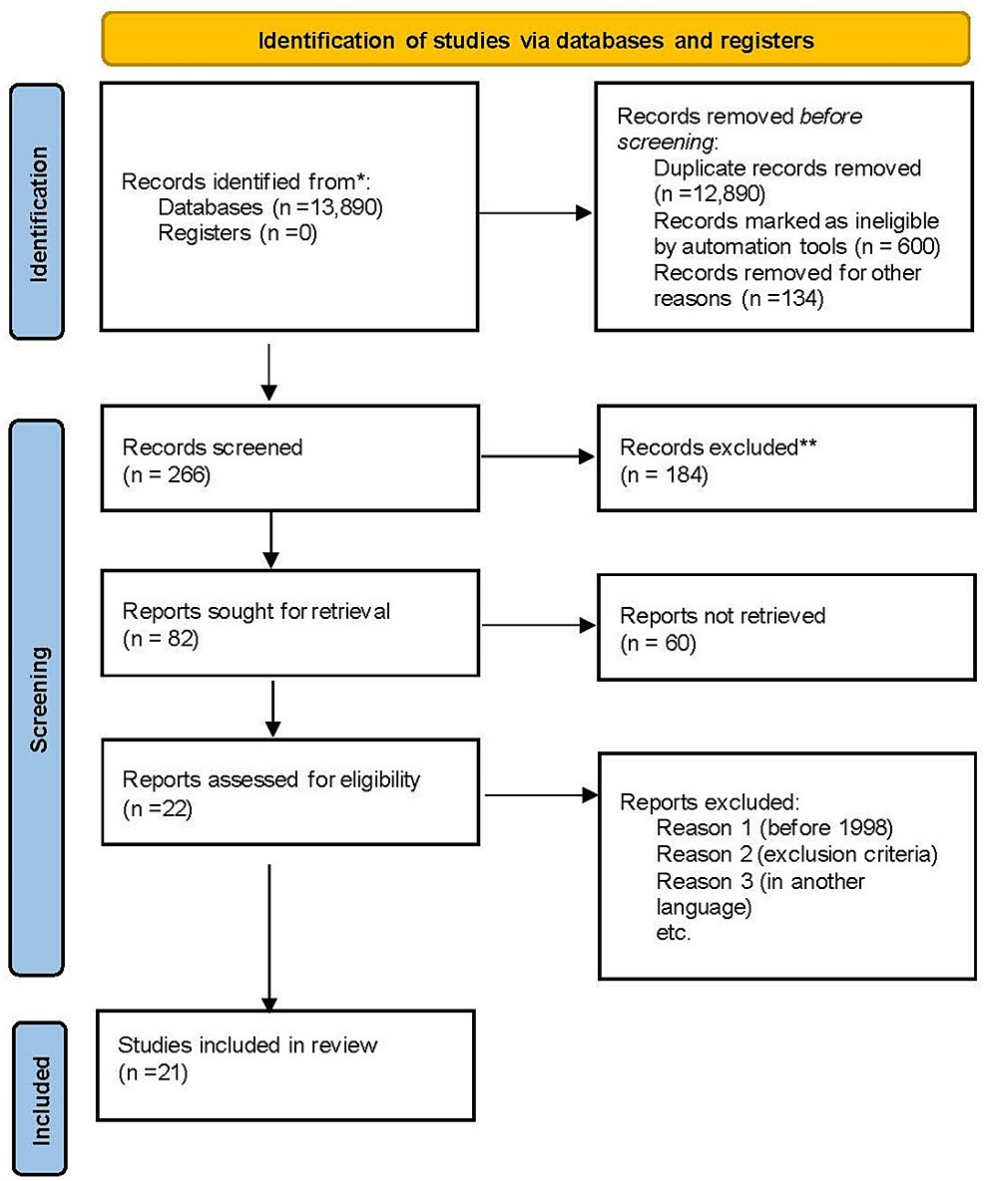
PRISMA flow diagram. PRISMA: Preferred Reporting Items for Systematic Reviews and Meta-Analysis

A total of 13,890 records were identified from both Google Scholar and PubMed database (Table 1). We screened 266 records, of which 184 were excluded. After removing duplicates, 600 articles were considered ineligible by automation tools, and 134 were removed for other reasons. We were left with 82 reports for retrieval, 60 were not retrieved, leaving 22 articles. Subsequently, based on the exclusion criteria, we included 21 studies in our study. Figure 1 shows the PRISMA flow diagram.

Table 3 presents the characteristics of the studies included in this systematic review.

**Table 2:** Characteristics of the studies included in this systematic review ASD: autistic spectrum disorder; MMR: Measles, Mumps, Rubella vaccine; Tdap: Tetanus, Diphtheria, and Pertussis vaccine

Author	Type of the studies	Year of publication	Conclusion
Gurney et al. [1]	A cohort study	2003	We observed dramatic increases in the prevalence of ASD as a primary educational disability starting in the 1991-1992 school year
Davidson [2]	Traditional review	2017	The article presents a narrative of the origin of the myths around autism
Wakefield et al. [3]	Retracted paper	1998	The study identified an association between gastrointestinal disease and developmental regression in a group of previously normal children, which is generally associated with a possible environmental trigger
Wilson et al. [4]	Systematic Review	2003	The current literature does not suggest an association between ASD and MMR
DeStefano et al. [5]	Annual review	2019	The current literature does not suggest an association between ASD and MMR
Blaylock [6]	Traditional review	2008	There is considerable evidence implicating a connection between the current vaccine schedule and the development of ASD
Hviid et al. [7]	Cohort study	2019	The study strongly supports that MMR vaccination does not increase the risk for autism
Hviid et al. [9]	Cohort study	2003	The results do not support a causal relationship between childhood vaccination with thimerosal-containing vaccines and the development of ASD
Gillber and Heijbel [10]	Case series	1998	MMR coverage substantially increased in the 1980s. ASD: 62% of the sample (34 children) were born before an increase in MMR vaccine coverage (55% of the period); 38% of the sample (21 children) were born after the rise in MMR coverage (45% f the sample)
Patja et al. [11]	Case series	2000	No cases of post-vaccination ASD (based on passive reporting) in about 1.8 million vaccinees
Peltola et al. [12]	Case series	1998	No cases of ASD in these children are after a mean follow-up of nine years and three months old
Taylor et al. [13]	MMR: coverage substantially increased in the 1980s. ASD: 62% of the sample (34 children) were born before the increase in MMR vaccine coverage (55% of the period); 38% Of the sample (21 children) were born after the increase (45% of the sample)	1999	MMR: introduced in 1987. ASD: No sudden step-up in cases of core and atypical autism in 1987 (p > 0.25); no change in trend in ASD before and after 1987
Farrington et al. [14]	Self-matched case series: compared rates of regression, parental concern, or diagnosis of ASD in specified periods after vaccination to all other periods for that individual (extended analysis of Taylor et al.)	2001	No increased incidence of diagnosis of ASI, regression, or parental concern 24, 36, or 60 months after vaccination; no increased likelihood of ASD, regression, or parental anxiety after immunization compared with before immunization
Dales et al. [15]	Time-series: compared increasing rates of ASD to rising rates of MMR vaccine coverage (1980–1994 birth cohorts)	2001	MMR: increase coverage from 72% to 82% (14% relative increase). ASD: increase in ASD births from 44/100,000 to 208/100,000 (373% relative increase)
Kaye et al. [16]	Time-series: compared increasing rates of ASD to changes in rates of MMR vaccine coverage	2001	MMR: rates stable at 97%. ASD: increase in cumulative incidence of ASD from 8/10,000 to 29/10,000 (p < 0.0001 trend)
DeWilde et al. [17]	Case-control study: compared changes in the number of consultations six months before to six months after the MMR vaccine as administered in autistic patients and control subjects	2001	No significant difference in change in the number of consultations between autistic patients (0.69 consultations per patient decrease) and controls (0.73 consultations per patient decrease) (p = 0.69); only one case of ASD was diagnosed within six months of MMR vaccination
Fombonne and Chakrabarti [18]	Cross-sectional study	2001	The rate of any developmental regression reported in the pre-MMR sample was 18.4% (P > .IS); in the post-MMR sample, 15.6%
Taylor et al. [19]	Time-series study	2002	MMR: introduced in October 1998. ASD: no trend in increasing percentages of children with ASD had bowel symptoms (OR = 0.98; 95% Cl = 0.93-1.04; p = 0.50) or who had regression (Cl = 0.93-1.03; p = 0.47) over the entire period
Madsen et al. [20]	Retrospective cohort: Determined if an association existed between the development of ASD and age at MMR vaccination or interval since MMR vaccination	2002	No association between the development of ASD and age at MMR vaccination (p = 0.23) or interval since MMR vaccination (p = 0.42)
Mäkelä et al. [21]	Retrospective cohort: determined if there is a clustering of hospitalizations for autism after the time of MMR vaccination	2002	Of 535,544 MMR vaccines, there were 309 hospitalized for autism after MMR vaccination. No clustering of hospitalizations was detected in the interval from MMR vaccination to hospitalization
Becerra-Culqui et al. [22]	Cohort study	2018	Antenatal Tdap vaccination is not associated with an increased ASD risk

Outcomes

In total, 19 articles were on the link between immunization and the incidence of autism. One article discussed the prevalence of ASD, the other about the effect of multiple immunizations during brain development (this study had data supporting the link between the current vaccine schedule and the development of ASD). The results of the 19 articles do not support a causal relationship between childhood immunization and the development of autism.

Discussion

ASD is a collection of phenotypic and developmental disorders resulting in significant social, communicative, and behavioral challenges. It is characterized by speech, language, and social functioning deficiencies and atypical behavioral symptoms, such as habitual, repetitive movements and extreme distress from environmental changes. Comorbidities include mental retardation, epilepsy, chronic gastrointestinal (GI) problems, and hyperactivity in certain persons. Parents of children with ASD frequently notice developmental difficulties during their child’s first year of life. The disorder has a vital genetic component [1,5]. Many conditions that were formerly diagnosed as separate entities are now included in ASD diagnosis. These include Asperger syndrome, pervasive developmental disorder (PDD) not otherwise specified, and autistic disorder. ASD with regression is a subtype where patients with ASD have lost previously gained developmental skills, most commonly for language. Unfortunately, there is no cure or single diagnostic approach for the disorder, although some data suggest that early, intensive behavioral therapy may improve functioning [5].

In the late 1990s, Andrew Wakefield, a physician at London’s Royal Free Hospital, published a paper in The Lancet claiming to have discovered the measles virus as the cause of autism. Initially, Wakefield stated that the measles virus caused colonic lesions in Crohn’s disease. Although this idea was quickly debunked and dismissed, Wakefield was impressed by cases brought to his attention in which typically developing children developed autistic symptoms after receiving the MMR triple vaccine. Despite his prior miscalculation with Crohn’s disease, he believed the measles virus had caused inflammatory lesions in the colon. All eight autistic patients on whom he had performed lower GI studies developed the hypothesized lesions, concluding that the measles vaccine virus led to the development of autism [2]. Wakefield’s findings could have raised warning lights at this time, even if the measles virus turned out to be a coincidental cause of autism. Wakefield’s claim was rapidly followed by reports of the detection of the measles virus in intestinal biopsies, blood, and cerebral spinal fluid samples taken from autistic children. After securing a straightforward and catchy scientific explanation, politicians and leaders of major groups of families of autistic children stood up with Wakefield.

In 2005, an investigative reporter brought to the attention of The Lancet’s editors that Wakefield’s study had been questioned by severe research misconduct, conflict of interests, and probably mendacity. After an inquiry into the subject, The Lancet withdrew the article, and the British Medical Association took strict actions against Wakefield. Since the Wakefield paper, any attempt to relate autism and the MMR vaccine has been disproved by many studies investigating the epidemiology of autism and the biological effects of MMR and the mumps virus. Mumps viruses were not regularly discovered in autistic children’s natural materials at a higher incidence than in non-autistic youngsters. Furthermore, there was no evidence that a decrease in the rate of MMR exposure was associated with reductions in the incidence of autism. Regardless of scientific data, it was assumed that if the mumps virus was not to blame for autism, another MMR ingredient must be [2]. The substantial rise in the incidence of ASD has sparked widespread public concern regarding the disorder’s causes and how to prevent them [1]. Despite extensive research about the etiology and pathophysiology of autism, few conclusions have been reached regarding a fundamental causal mechanism. There is no cure currently.

Several hypotheses were posited on the relationship between vaccination and autism development. The first theory relates to immune system dysfunction, organic acid synthesis, the effects of gliamorphin on cerebral function, and mercury toxicity. Parents are concerned about the safety of their children receiving numerous immunizations simultaneously. According to this theory, frequent stimulation of the systemic immune system by vaccination causes a strong microglial reaction in the growing brain, leading to changes in immunological function, resulting in synaptic, dendritic loss, and aberrant appearance pathways. When the microglia are activated, the brain’s immune cells release inflammatory cytokines, free radicals, lipid peroxidation products, and two excitotoxins: glutamate and quinolinic acid. Consequently, clinical and pathological characteristics of autism emerge. Microglia are also activated by mercury at quantities of less than 0.5 µg (3 to 5 ng) per gram of moist tissue. High mercuric products are toxic to both the kidneys and the brain. Thimerosal is an organic chemical that includes ethyl mercury and forms a preservative in vaccines. Ethyl mercury hydroxide quickly penetrates the brain and converts to inorganic mercury [6].

Hviid et al. [9] compared children who received immunization with a thimerosal-containing pertussis vaccine to children vaccinated with the same pertussis vaccine formulated without thimerosal and followed them for the symptoms and the signs of autism and other ASD. It was a population-based cohort study to identify the association between thimerosal and autism. In Denmark, they found that the risk of autism and other ASD did not vary greatly between children immunized with a thimerosal-containing vaccine and children immunized with the thimerosal-free vaccine (relative risk (RR) = 0.85 [95% confidence interval (CI) = 0.60-1.20] for autism; RR = 1.12 [95% CI = 0.88-1.43] for other ASD). They also highlighted that there was no proof of a dose-response correlation (increase in RR per 25 µg of ethyl mercury = 0.98 [95% CI = 0.90-1.06] for autism and 1.03 [95% CI = 0.98-1.09] for ASD) [9].

The second hypothesis states that the MMR vaccine can cause autism. Measles is an exceptionally contagious viral infection caused by a paramyxovirus (genus Morbillivirus). It is disseminated through the respiratory system. The clinical features start with a prodrome of flu-like illness, followed by cough, coryza, and conjunctivitis. The measles rash appears as a maculopapular rash on the head that spreads to the torso and arms and legs over three to four days (Koplik spots), which are blue-white plaques on the mucous membranes of the mouth that are pathognomonic. Possible complications include otitis media, viral or bacterial pneumonia, visual loss, acute encephalitis, seizures, and death. Measles is still a significant reason for death and disability in developing countries.

Mumps is a viral infection caused by a paramyxovirus (genus Rubulavirus) transmitted through the respiratory system. Mumps, or measles, virus infection usually results in permanent immunity. Parotitis appears 16-18 days after exposure, and most patients are asymptomatic. Orchitis is more prevalent in post-pubertal boys. Mumps complications are rare and most common in adults, including aseptic meningitis, encephalitis, pancreatitis, and deafness [5].

Rubella (German measles) is another viral infection distributed through the respiratory system. It is caused by togavirus (genus Rubivirus). Fever, malaise, upper respiratory symptoms, and a maculopapular rash are the signs and symptoms of rubella, which appear 14 days after contact. Rubella complications are uncommon, although more common in adults and older children. Congenital rubella syndrome (CRS) is a condition that affects the developing fetus and is more severe when infection occurs early in pregnancy. The rubella virus causes fetal death, early birth, deafness, blindness, and severe birth problems, and infection with the virus usually results in lifetime immunity [5].

MMR Vaccine

The MMR vaccine is part of the required childhood vaccination schedule in the United States. It is given in two doses, the first at 12-15 months and the second at 4-6 years. MMR vaccines are live attenuated virus vaccines to prevent measles (rubeola), mumps, and rubella. It is well-tolerated. The most common side effects are injection site responses, fever (5-15%), and a minor rash (5%). The first shot of the MMR vaccine is associated with afebrile seizures. Thrombocytopenia (low platelet count) is a rare yet severe side effect that happens in roughly 1 in 30,000-40,000 doses. Measles vaccines were available in the United States from the beginning to the mid-1960s, and the MMR vaccine was introduced in 1971. Despite a recurrence of measles in the United States and worldwide from 1989 to 1991, cases continued to drop, and measles was declared eradicated in the United States in 2000.

Mumps, measles, rubella and CRS cases have had a dropdown in the United States since the introduction of the MMR vaccine [5]. Wakefield et al. published a report in 1998 describing 12 cases of pervasive developmental delay and developmental regression linked to GI tract symptoms and developmental regression, many of which occurred shortly after the patient received the MMR vaccine. This case study suggests that a new type of ASD was emerging and linked to the MMR vaccine. Although the study’s methodologies were extensively questioned, it raised considerable public worry about the MMR vaccine’s safety. Several epidemiological studies have been conducted to understand the association between ASD and the MMR vaccine. These studies have been designed to study multiple hypotheses put forth by the report of Wakefield et al. and others that have advised against using the MMR vaccine. The specific hypotheses that have been studied are (1) rates of ASD are increasing in people who have been given the MMR immunization than in those who have not, (2) a rise in ASD may be occurring as a consequence of the MMR vaccine, (3) the development of ASD is being momentarily linked to receiving the MMR vaccine, and (4) the MMR vaccine may be linked to a new variant form of ASD [4,5].

The tables below will summarize and present the results of these studies; it does not suggest a correlation between ASD and the MMR vaccine [4]. Table 4 shows the characteristic of these studies.

**Table 3:** Characteristics of the included studies. ASD: autistic spectrum disorder; DSM Ill-R: Diagnostic and Statistical Manual of Mental Disorders, Revised Third Edition; DSM IV: Diagnostic and Statistical Manual of Mental Disorders, Revised Fourth Edition; Gl: gastrointestinal; ICD: International Classification of Diseases; MMR: measles-mumps-rubella *This is the population studied for analysis data that we extracted; ^#^Same dataset as Peltola et al. [12] was used; ^+^same dataset as Taylor et al. [13] was used.

Source	Country	Population*	Mechanism of ASD diagnosis
Gillber and Heijbel [10], 1998	Sweden	A population study of children born between 1975 and 1984 diagnosed with ASD in Goteberg and Bohuslan, Sweden (N = 55)	Diagnosis of DSM III-R autistic disorder by a team of experts
Patja et al. [11], 2000#	Finland	All children receiving MMR vaccinations between 1982 and 1996 (about 1.8 million vaccinees)	Passive reporting of adverse events to National Public Health Institute Report by healthcare providers
Peltola et al. [12],1998	Finland	31 children reported having developed Gl tract symptoms after receiving an MMR vaccination	Based on a review of hospital or health center records or interviews with public health nurses (Mean of nine years and three months after GI tract symptoms developed)
Taylor et al. [13], 1999	UK	Children younger than 16 years, born from 1979 to mid-1998, with ASD in 8 health districts (498 children with ASD: 261 with core ASD, 166 with atypical ASD, 71 with Asperger syndrome)	Computerized, special needs or disability registers at child development centers, records in special schools (checked by pediatric registrars using ICD-10 classification)
Farrington et al. [14], 2001*	UK	Extended analysis of Taylor et al. 1999 (n = 357 for diagnosis of ASD, 326 for parental concern, and 105 for regression)	Child development centers and special schools (checked by pediatric registrars using ICD-10 classification)
Dales al. [15], 2001	USA	Statewide surveys (California): random samples of kindergarten pupils, immunization records at 24 months of age born between 1980 and 1994 (600-1,900/y)	ASD caseload of the department of Developmental Services Regional Centers for persons with disabilities (ICD-9 classification)
Kaye et al. [16], 2001	UK	Consecutive annual birth cohorts of autistic boys born during 1988–1993 (114 autistic boys, aged 2–5 years)	UK General Practice Database (general practitioner diagnosis with 81% referred to specialists)
DeWilde et al. [17], 2001	UK	71 children with ASD, 284 matched controls; identified from UK General Practice Database between 1989 and 2000	General practitioner diagnosis
Fombonne and Chakrabarti [18], 2001	UK	(1) Epidemiological sample of 96 children with pervasive developmental disorder born between 1992 and 1995 (after the introduction of the MMR vaccine). After the introduction of the MMR vaccine, the clinical sample of 68 autistic children born between 1987 and 1996. Before the introduction of the MMR vaccine, a clinical sample of 98 autistic children born between 1954 and 1979	Autism diagnostic interview-revised and ICD-10/DSM-diagnosis
Taylor et al. [19], 2002*	UK	Children younger than 16 years, born from 1979 to mid-1998, with ASDs in 8 health districts (473 children with autism: 278 with core autism. and 195 with atypical autism)	Computerized, special needs/disability registers at child development centers, records in special schools, child psychiatric records (checked by pediatric registrars using ICD-10 classification)
Madsen et al. [20],2002	Denmark	All children born between January 1991 and December 1998 and registered in the Danish Civil Registration System: vaccination data based on general practitioners’ reports to the National Board of Health	All diagnoses in hospitals and outpatient clinics based on ICD-10 codes were identified from Danish Psychiatric Central Register; a child psychiatrist reviewed 40 charts for confirmation
Makela et al [21], 2002	Finland	535,544 vaccinees aged 1 to 7 years enrolled in a surveillance study between November 1982 and June 1986	Hospitalizations for autism based on ICD-8 or 9 codes from the nationwide hospital register between November 1982 and December 1995

Table 5 shows the comparison of the rate of ASD in vaccinated and unvaccinated Individuals.

**Table 4:** Comparison of the rate of ASD in vaccinated and unvaccinated individuals. The table shows no statistically significant differences in the rates of autism or ASD between these two populations in adjusted and non-adjusted analyses [4]. ASD: autistic spectrum disorder; RR: relative risk; CI: confidence interval

Source	Analysis	Finding
Madsen et al. [20] 2002	Determining the rates of autism and ASD in 440,655 vaccinated and 96,648 unvaccinated individuals; analysis was based on person-years of follow-up and was adjusted on confounding variables	Adjusted RR of autism in vaccinated individuals = 0.92 (95% Cl = 0.68-1.24); adjusted RR of ASD in vaccinated individuals = 0.83 (95% CI = 0.65-1.07)

Table 6 compares the changes in the rate of ASD with changes in MMR vaccine coverage.

**Table 5:** Comparison of the changes in the rate of ASD with change in MMR vaccine coverage. It did not find an increase in ASD rates in the period of MMR vaccination [4]. ASD: autistic spectrum disorder; MMR: Measles, Mumps, Rubella vaccine; GI: gastrointestinal; OR: odds ratio; CI: confidence interval

Source	Analysis	Findings
Gillber and Heijbel [10], 1998	Case series: compared proportions of autistic cases in high and low coverage periods	MMR: coverage substantially increased in the 1980s ASD: 62% of the sample (34 children) were born before the increase in MMR vaccine coverage (55% of the period); 38% of the sample (21 children) were born after the increase in MMR coverage (45% of the sample)
Taylor et al. [13], 1999	Time-series: compared changes in the rates of ASD in periods before and after the MMR vaccine was introduced	MMR: introduced in 1987. ASD: no sudden step-up in cases of core and atypical autism in 1987 (p > 0.25); no change in the trend of ASD before and after 1987
Dales et al. [15], 2001	Time-series: compared increasing rates of ASD to rising rates of MMR vaccine coverage (1980–1994 birth cohorts)	MMR: increase in coverage from 72% to 82% (14% relative increase). ASD: increase in ASD births from 44/100,000 to 208/100,000 (373% relative increase)
Kaye et al. [16], 2001	Time-series: compared increasing rates of ASD to changes in the rates of MMR vaccine coverage	MMR: rates stable at 97%. ASD: increase in cumulative incidence of ASD from 8/10,000 to 29/10,000 (p < 0.0001 trend)
Fombonne and Chakrabarti [18], 2001	Cross-sectional study: compared rates of developmental regression in samples of autistic children before and after the introduction of the MMR vaccine	The rate of any developmental regression reported in the pre-MMR sample was 18.4% (p > .IS); in the post-MMR sample, 15.6%
Taylor et al. [19], 2002	Time-series: determined if there was an increasing percentage of children with ASD and either Gl tract symptom or regression between 1979 and 1998	MMR: introduced in October 1998. ASD: no trend in increasing percentages of children with ASD who had bowel symptoms (OR = 0.98; 95% Cl = 0.93-1.04; p = 0.50) or who had regression (OR Cl = 0.93-1.03; p = 0.47) over the entire period

Table 7 shows the temporal association of ASD with the MMR vaccine.

**Table 6:** Temporal association of ASD with the MMR vaccine. ASD: autistic spectrum disorder; MMR: Measles, Mumps, Rubella vaccine [4]

Source	Analysis	Findings
Taylor et al. [13], 1999	Case series: compared ages of ASD diagnosis in those vaccinated before 18 months, after 18 months, and those not vaccinated. Self-matched case series: compared rates of regression, parental concern, or the diagnosis of ASD in specified periods after vaccination	There was no significant difference in age at diagnosis among the three groups (p = 0.41). No increased incidence of diagnosis of ASD or regression six months and one year after vaccination; significantly increased risk of parental concern six months after vaccination (p = 0.03)
Patja et al. [11], 2000	Case series: identified all reports of vaccine-related complications	No cases of post-vaccination ASD (based on passive reporting) in about 1.8 million vaccinees
Farrington et al. [14], 2001	Self-matched case series	No increased incidence of diagnosis of ASD, regression, or parental concern (24, 36, or 60 months) after vaccination; no increased likelihood of ASD, deterioration, or parental anxiety after immunization compared with before immunization
DeWilde et al. [17], 2001	Case-control study	No significant difference in change in the number of consultations between autistic patients (0.69 consultations per patient decrease) and controls (0.73 consultations per patient decrease) (p = 0.69); only one case of ASD was diagnosed within six months of MMR vaccination
Fombonne and Chakrabarti [18], 2001	Cross-sectional study: compared ages of first parental concern between population samples exposed to MMR vaccine and a pre-MMR vaccine sample. The compared mean interval from MMR to parental anxiety in autistic children with and without regression	Mean ages of first parental concern in post-MMR vaccine samples were 19.3 and 19.2 months (in pre-MMR samples) and 19.5 months (p > 0.05). The mean interval in patients with ASD who had regression was 248 days; in patients with ASD who did not deteriorate, 272 days (p > 0.05)
Taylor et al. [19], 2001	Case series: determined whether vaccine received before the development of parental concern, after the development of anxiety, or not in autistic children with Gl tract symptoms or developmental regression	Gl tract symptoms: 19% received MMR vaccine before parental concern, 15% after concern, and 16% did not receive MMR vaccine (p = 0.48). Regression: 26% received MMR vaccine before parental concern, 26% after concern, and 30% did not receive the vaccine (p = 0.83)
Madsen et al. [20], 2002	Retrospective cohort	There is no association between ASD development and the age at MMR vaccination (p = 0.23) or interval since MMR vaccination (p = 0.42)
Makela et al. [21], 2002	Retrospective cohort	Of the 535,544 MMR vaccines, there were 309 hospitalized for autism after MMR vaccination; no clustering of hospitalizations was detected (in the interval from MMR vaccination to hospitalization)

Table 8 shows the specific association between variant autism and the MMR vaccine.

**Table 7:** Specific association of variant autism and the MMR vaccine. GI: gastrointestinal; ASD: autistic spectrum disorder; MMR: Measles, Mumps, Rubella vaccine; OR: odds ratio; CI: confidence interval [4]

Source	Analysis	Findings
Peltola et al. [12] 1998	Case series: conducted follow-up of 31 vaccinated children reported to have Gl tract symptoms (of about 3 million vaccine doses)	No cases of ASD in these children (after a mean follow-up of nine years and three months)
Fombonne and Chakrabarti et al. [18] 2001	Cross-sectional study: compared rates of the developmental regression in samples of autistic children before and after the introduction of the MMR vaccine. The compared mean interval from MMR to parental concern in autistic children with and without regression. Determined rates of childhood disintegrative disorder after the introduction of the MMR vaccine sample	Rate of any developmental regression reported in post-MMR sample = 15.6%; in pre-MMR sample = 18.4% (p > 0.15) Mean interval in patients with ASD and regression, 248 days: in patients with ASD who did not have regression, 272 days (p > 0.05). Low incidence of childhood disintegrative disorder in the epidemiological sample after MMR vaccination (0.6/10,000)
Taylor et al. [19] 2002	Time-series: identified if there was an increasing percentage of children with ASD and either Gl tract symptoms or regression between 1979 and 1998 (MMR vaccine introduced in 1998). Case series: determined whether MMR vaccine was received before the development of parental concern, after the development of concern, or not at all in autistic children with Gl tract symptoms or developmental regression	No increase in percentages of children with ASD who had either bowel symptoms (OR = 0.98; 95% Cl = 0.93-1.04; p = 0.50) or who had regression (OR = 0.98; Cl = 0.93-1.03; p = 0.47) over the entire period of Gl tract symptoms: 19% received MMR vaccine before parental concern, 15% after concern, and 16% did not receive MMR vaccine (p = 0.48). Regression: 26% received MMR vaccine before parental concern, 26% after concern, 30% did not receive MMR vaccine (p = 0.83)
Makela et al. [21] 2002	Retrospective cohort: determined if any recipients of MMR vaccines hospitalized with autism were also hospitalized with inflammatory bowel disease	No hospital visits for inflammatory bowel disease among 309 children hospitalized with autism

Hviid et al. conducted a nationwide cohort review of all infants born in Denmark to Danish-born mothers from January 1, 1999, through December 31, 2010, to see whether MMR immunization carries a high risk for autism in children, subgroups of children, or periods after vaccination. In Denmark, 657,461 babies born between 1999 and December 31, 2010, participated, with follow-up from one year of age to August 31, 2013 (Danish Civil Registration System is the source of patient information). They found no support for high autism risk after MMR vaccination in a national broad, unselected cohort population of Danish children. In a 2014 meta-analysis of MMR vaccine and autism studies, researchers found two cohorts and four case-control studies from Denmark, Poland, Japan, the United Kingdom, and the United States, with no evidence of a link, for example, a pooled odds ratio from cohort studies of 0.84 (CI = 0.70 to 1.01) [2,4,5,7].

The third hypothesis claims that antenatal Tdap vaccination is linked to a higher risk of ASD. Becerra-Culqui et al. examined the link between antenatal tetanus, diphtheria, and acellular pertussis (Tdap) vaccination and the offspring’s risk of ASD. With the rise in the frequency of ASD and the increased vaccination in pregnant women, it is more vital than ever to analyze the safety risks associated with prenatal immunization. This study is a retrospective cohort study of mother-baby pairs that gave birth at Kaiser Permanente Southern California hospitals between January 1, 2011, and December 31, 2014. They used digital medical data to get maternal Tdap immunization from pregnancy to the delivery date. The International Classification of Diseases, Ninth, and Tenth Revision codes conveyed an ASD diagnosis. Children were cared for from birth until their first ASD diagnosis, the end of their membership, or their follow-up (June 30, 2017). According to this study, prenatal Tdap immunization was not linked to an increased incidence of ASD. Table 9 shows the frequencies and associations between Tdap vaccination during pregnancy and ASD in infants born between 2011 and 2014.

**Table 8:** Frequencies and associations between Tdap vaccination during pregnancy and ASD in infants born between 2011 and 2014. IPTW: inverse probability of treatment weighting; HR: hazard ratio; CI: confidence interval. Adjustments were made for the child’s birth year, gestational age at birth (<37 or ≥37 weeks); maternal age, race and ethnicity, and education; Medicaid insurance, medical center of delivery, parity, the start of prenatal care, and influenza vaccination during pregnancy [22].

	ASD incidence rate per 1,000 person-years		HR (95% CI)	
	Unvaccinated	Vaccinated	Unadjusted	IPTW-adjusted
Overall	4.05	3.78	0.98 (0.88–1.09)	0.85 (0.77–0.95)
Birth year				
2011	3.57	3.22	0.91 (0.74–1.12)	0.86 (0.70–1.07)
2012	4.02	3.18	0.80 (0.62–1.02)	0.80 (0.63–1.03)
2013	4.48	4.46	1.00 (0.81–1.23)	0.99 (0.80–1.23)
2014	4.87	4.14	0.89 (0.68–1.18)	0.85 (0.65–1.12)
Nulliparous	4.88	4.56	0.99 (0.85–1.15)	0.75–1.02)

## Conclusions

According to our review, there is no link between the development of ASD and immunization. The dramatic increase in the prevalence of ASD created widespread concern. Many theories have been offered to explain the link between vaccination and the development of autism, including changes in immune system function, abnormal organic acid synthesis, mercury toxicity, the effects of gliamorphin on cerebral function, and the link between MMR and autism. However, all these theories remain theoretical, and our review finds no evidence of a link between them and the development of autism. Parents experienced vaccination reluctance following the release of the Wakefield study on the supposed MMR vaccine-autism relationship. It raises concern and challenges vaccine acceptance among parents, leading to the re-emergence of vaccine-preventable diseases. It still raises concern in some parents; we recommend that public health officials continue to advocate and encourage vaccination. The public may require more studies to rule out the association between ASD and vaccination.
